# Pre-existing Proton Pump Inhibitor Treatment and Short-Term Prognosis of Acute Myocardial Infarction Patients

**DOI:** 10.3389/fcvm.2022.919716

**Published:** 2022-07-04

**Authors:** Juntao Xie, Qingui Chen, Dejian He

**Affiliations:** ^1^Intensive Care Unit, The First People’s Hospital of Chenzhou, Chenzhou, China; ^2^Intensive Care Unit, The Chenzhou Affiliated Hospital, Hengyang Medical School, University of South China, Chenzhou, China; ^3^Department of Medical Intensive Care Unit, The First Affiliated Hospital, Sun Yat-sen University, Guangzhou, China; ^4^Department of Emergency, The First People’s Hospital of Chenzhou, Chenzhou, China; ^5^Department of Emergency, The Chenzhou Affiliated Hospital, Hengyang Medical School, University of South China, Chenzhou, China

**Keywords:** myocardial infarction, proton pump inhibitors, histamine 2 receptor antagonists, risk factors, prognosis

## Abstract

**Introduction:**

Evidence suspects proton pump inhibitor (PPI) use is a risk factor of poor prognosis of acute myocardial infarction (AMI). We aimed to investigate the association between pre-existing PPI use before emergency department (ED) visit and short-term prognosis of AMI patients.

**Materials and Methods:**

AMI patients admitted to ED were included and categorized as cohorts with or without pre-existing PPI use. Hospital mortality, length of hospital stay, being admitted to intensive care unit (ICU), and length of (total) ICU stay were studied as prognostic outcomes. Multivariable logistic regression or linear regression were used to estimate the associations between pre-existing PPI use and the outcomes after adjusting for potential confounders.

**Results:**

A total of 2001 AMI patients were included. No significant difference was found in hospital mortality and length of ICU stay between cohorts; patients with pre-existing PPI use showed a significantly longer length of hospital stay (median 3.81 vs. 3.20 days, *P* = 0.002) but lower proportion of being admitted to ICU (25.59% vs. 40.83%, *P* < 0.001) compared to those without pre-existing PPI use. Pre-existing PPI use was not associated with hospital mortality [odds ratio (OR) 1.08, 95% confidence interval (CI) 0.58–1.99], length of hospital stay (β = 0.23, 95% CI −0.35 to 0.82), and length of ICU stay (β = −0.18, 95% CI −1.06 to 0.69), but was statistically significantly associated with lower risk of being admitted to ICU (OR 0.69, 95% CI 0.52–0.92).

**Conclusion:**

The current study does not support newly diagnosed AMI patients with pre-existing PPI use before ED visit would experience worse short-term prognosis than those without.

## Introduction

Proton pump inhibitor (PPI) is a class of medications that effectively blocks gastric acid secretion *via* inhibition of the gastric hydrogen-potassium ATPase (1, 2). Currently, the indications of PPI treatment include peptic ulcer disease, gastroesophageal reflux disease, Zollinger-Ellison syndrome, non-steroidal anti-inflammatory drug (NSAID)-associated ulcers, and the eradication of Helicobacter pylori (1, 3). Compared to another type of antisecretory agent histamine 2 receptor antagonists (H_2_RA), PPI shows faster control of peptic ulcer disease symptoms, higher ulcer healing rates (4–6), and better effectiveness in preventing or healing NSAID-associated ulcers (7). This is consistent with the increasing prescription of PPI observed in daily practice during the past three decades (8). However, it has been observed that long-term use of PPI is associated with increased risk of some adverse events, including Clostridioides difficile and other enteric infections (9–11), microscopic colitis (12), intestinal colonization of multi-drug resistant organisms (13), malabsorption of minerals and vitamins (14, 15), pneumonia (16–19), and mortality (20), and such safety concerns are deepened with the observed overutilization of PPI (21). It should be noted that few of the above associations have been convincedly proved to be causal (22), which, instead, may be mainly due to uncontrolled confounding.

Acute myocardial infarction (AMI) is a rather common severe condition (23) and exposure to PPI is suspected to increase the risk of major adverse cardiovascular events including AMI in the general population (24, 25) [although results are inconsistent between studies (26)], while the role of PPI use in AMI patients has not been well established (27). A meta-analysis with a sample size of 33,492 patients found concomitant PPI was associated increased risk of major adverse cardiovascular events in patients taking aspirin and clopidogrel (28), while results from a recent meta-analysis with a sample size of 190,476 patients did not support such an association in patients treated with dual antiplatelet therapy after percutaneous coronary intervention or acute coronary syndrome (29). Nevertheless, the existing evidence is only about PPI use which was started after AMI had been established, but little is unknown about whether newly diagnosed AMI patients with pre-existing PPI use would experience worse prognosis than those without. Given the wide use of PPI (21), AMI patients with pre-existing PPI use may account for a substantial proportion, and thus it is clinically relevant to examine the prognosis of this subgroup of AMI patients. The current study aimed to investigate the association between pre-existing PPI use before emergency department (ED) visit and short-term prognosis of AMI patients.

## Materials and Methods

### Data Source

In the current study we used data from the Medical Information Mart for Intensive Care (MIMIC)-IV (version 1.0) (30) and its module the MIMIC-IV-ED database (version 1.0) (31). In brief, the MIMIC-IV database contains medical records (including vital signs, laboratory measurements, diagnosis, administered medications during the hospitalizations) of patients who were admitted to one of the intensive care units (ICUs) or the ED at the Beth Israel Deaconess Medical Center (BIDMC, a tertiary academic medical center in Boston, United States) between 2008 and 2019; the MIMIC-IV-ED database contains medical records in the ED at the BIDMC between 2011 and 2019. These two databases were created under the Health Insurance Portability and Accountability Act (HIPAA) safe harbor provision after approved by the Institutional Review Boards (IRBs) of Beth Israel Deaconess Medical Center, Boston, MA, and the Massachusetts Institute of Technology, Cambridge, MA (#2001P001699) (30, 31).

According to the required procedures of requesting access to the databases, we first completed the Collaborative Institutional Training Initiative (CITI) “Data or Specimens Only Research” course and then applied for the access, which was further approved by the database administrator. Since all the data in the databases are de-identified, the current study did not constitute research with human subjects given the nature of a secondary use of existing de-identified data in which there was no interaction with any individual and no identifiable private information was used. For this reason, the current study was exempted from further IRB approval and patient consent was waived, which was approved by the ethics committee of The First People’s Hospital of Chenzhou. When conducting the current study, we complied with the Helsinki Declaration 1964, and its later amendments. Detailed description of the databases could be found according to the attached references (30, 31).

### Study Population

We included patients who were admitted to the ED of the BIDMC between 2011 and 2019 with a primary diagnosis of AMI. AMI was identified by International Classification of Diseases, Ninth Revision, Clinical Modification (ICD-9-CM) code “410” (including any codes started with 410) or International Classification of Diseases, Tenth Revision, Clinical Modification (ICD-10-CM) code “I21” (including any codes started with I21, Supplementary Table 1). A primary diagnosis was identified by the sequence of diagnoses made during the ED stay in which the first diagnosis was considered as the primary diagnosis. Patients without medical records in the MIMIC-IV database were excluded due to the lack of hospitalization record.

### Exposure and Outcomes

The included patients were further categorized as two cohorts according to whether they were with pre-existing PPI use before ED visit. This was identified by the data about medicine reconciliation routinely collected on ED admission. In brief, when a patient was admitted to the ED, staffs would ask the patient what current medications he/she was taking. We used the key word “proton pump inhibitors” to identify this exposure, because this key word appeared in all records related to PPI in the database. Outcomes of the current study included hospital mortality, length of hospital stay, being admitted to ICU, and length of ICU stay. If a patient had multiple ICU admissions during the hospitalization, the length of ICU stay referred to the total length of all ICU stays during the hospitalization.

### Covariates

We extracted the below covariates in the study: age, sex, ethnicity; pre-existing H_2_RA use (before ED visit), detailed types of H_2_RA; cardiac biomarkers including troponin T and creatine kinase, MB isoenzyme (CK-MB); cardiac interventions during the hospitalization including percutaneous transluminal coronary angioplasty (PTCA), dilation of coronary artery, and coronary artery bypass graft (CABG); Charlson Comorbidity Index and various comorbidities including congestive heart failure, cerebrovascular disease, peripheral vascular disease, dementia, chronic pulmonary disease, rheumatic disease, peptic ulcer disease, mild liver disease, diabetes without complication, diabetes with complication, paraplegia, renal disease, malignant cancer, severe liver disease, metastatic solid tumor, and acquired immunodeficiency syndrome.

Pre-existing H_2_RA use was identified in a similar way to the identification of the exposure but using the key word ‘‘histamine H_2_-receptor antagonists.’’ The cardiac biomarkers referred to the maximum values within 24 h after admitted to the ED, which were identified by identifiers ‘‘51003’’ for troponin T and ‘‘50911’’ for CK-MB. The normal ranges of troponin T and CK-MB in the database were 0–0.01 ng/mL and 0–10 ng/mL, respectively. The cardiac interventions were identified according to key words of the procedures: ‘‘PTCA’’ for PTCA; ‘‘dilation of coronary artery’’ for dilation of coronary artery; and ‘‘bypass coronary artery,’’ ‘‘(aorto) coronary bypass,’’ and ‘‘single internal mammary-coronary artery bypass’’ for CABG. The Charlson Comorbidity Index and the various comorbidities were identified using codes from the code repository mimic-iv^1^ which was based on ICD-9-CM or ICD-10-CM according to diagnoses records during the hospitalization.

### Statistical Analysis

Summary statistics were presented as mean ± standard deviation or median (25th–75th percentile) for continuous variables according to whether they were normally distributed, and as number (percentage) for categorical variables. Comparisons between two groups were examined by the *t*-test or the Kruskal-Wallis H test for continuous variables, and by the Chi-squared test or the Fisher’s exact test for categorical variables. To evaluate the association between the exposure (i.e., pre-existing PPI use) and the study outcomes, we first performed univariable regression analysis to assess the association of the studied covariates with the outcomes, and then those covariates associated with the outcomes (identified by a *P*-value < 0.1) were included into the multivariable regression model to assess the association between the exposure and the outcomes. For the outcomes hospital mortality and being admitted to ICU, logistic regression was used; for the outcomes length of hospital stay and length of ICU stay, linear regression was used. Only patients with ICU admission during the hospitalization were included in the analysis of the outcome length of ICU stay. As an extra analysis, we categorized the study population as two cohorts according to whether they were with pre-existing H_2_RA use and evaluated the associations between pre-existing H2RA use and the study outcomes in the same way as the main analysis stated above. A *P*-value less than 0.05 was considered to indicate statistical significance. IBM SPSS Statistics for Windows (version 25.0. Armonk, NY: IBM Corp.) was used for the statistical analyses.

## Results

### Clinical Characteristics of the Study Population

**FIGURE 1:**
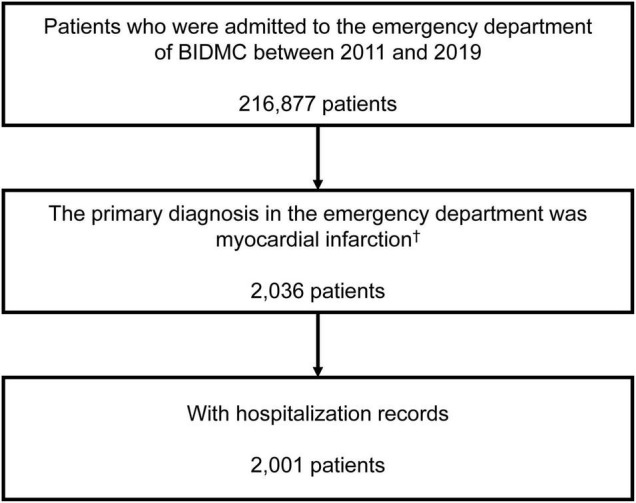
Inclusion of the study population. ^†^The first emergency department admission if a patient had more than one emergency department admission. BIDMC, Beth Israel Deaconess Medical Center.

We included a total of 2001 AMI patients finally (Figure 1). Among the included AMI patients, 44.3% (886/2001) were identified by ICD-9-CM codes and 55.7% were identified by ICD-10-CM codes. Detailed types of AMI were presented in Supplementary Table 1. The proportion of pre-existing PPI use was 21.3% (426/2001), and omeprazole was the most frequently prescribed PPI (67.1%, 286/426), followed by pantoprazole (28.4%, 121/426), esomeprazole (4.9%, 21/426) and rabeprazole (0.2%, 1/426).

Table 1 presented the clinical characteristics of the study population according to pre-existing PPI use. Compared to those without pre-existing PPI use, patients with pre-existing PPI use had a significantly older mean age (72.19 ± 12.91 vs. 68.94 ± 14.50 years, *P* < 0.001), lower proportion of male (52.58% vs. 61.02%, *P* = 0.002), and higher median Charlson Comorbidity Index [7 (5–9) vs. 5 (4–7), *P* < 0.001], but a lower median troponin T [0.33 (0.13–1.01) vs. 0.72 (0.23–2.34) ng/mL, *P* < 0.001] and CK-MB [10 (4.25–26.75) vs. 18 (6–67) ng/mL, *P* < 0.001], and lower proportions of receiving PTCA (13.62% vs. 21.97%, *P* < 0.001) or dilation of coronary artery (18.54% vs. 24.00%, *P* = 0.017). There was no statistically significant difference in the proportion of pre-existing H_2_RA use between the two cohorts. In terms of specific comorbidities, patients with pre-existing PPI use showed higher prevalence of congestive heart failure, chronic pulmonary disease, rheumatic disease, mild liver disease, diabetes with complication, renal disease, and severe liver disease compared to those without pre-existing PPI use.

**TABLE 1:** Clinical characteristics of the study population.

	Overall (*n* = 2,001)	Pre-existing use of proton pump inhibitor		P
		No (*n* = 1,575)	Yes (*n* = 426)	
Age (years)	69.63 ± 14.23	68.94 ± 14.50	72.19 ± 12.91	<0.001
Male	1,185 (59.22%)	961 (61.02%)	224 (52.58%)	0.002
Ethnicity				<0.001
White	1,424 (71.34%)	1,100 (69.93%)	324 (76.60%)	
Black/African American	227 (11.37%)	176 (11.19%)	51 (12.06%)	
Hispanic/latino	63 (3.16%)	47 (2.99%)	16 (3.78%)	
Asian	60 (3.01%)	47 (2.99%)	13 (3.07%)	
Other/unknown	222 (11.12%)	203 (12.91%)	19 (4.49%)	
Prior H_2_ receptor antagonist	94 (4.70%)	80 (5.08%)	14 (3.29%)	0.121
Prior famotidine	18 (0.90%)	17 (1.08%)	1 (0.23%)	0.101
Prior ranitidine	76 (3.80%)	63 (4.00%)	13 (3.05%)	0.364
Troponin T^†^ (ng/mL)	0.59 (0.20–1.91)	0.72 (0.23–2.34)	0.33 (0.13–1.01)	<0.001
CK-MB^†^ (ng/mL)	15 (6–54)	18 (6–67)	10 (4.25–26.75)	<0.001
PTCA	404 (20.19%)	346 (21.97%)	58 (13.62%)	<0.001
Dilation of coronary artery	457 (22.84%)	378 (24.00%)	79 (18.54%)	0.017
CABG	167 (8.35%)	134 (8.51%)	33 (7.75%)	0.614
Charlson comorbidity Index^‡^	6 (4–8)	5 (4–7)	7 (5–9)	<0.001
**Comorbidities^‡^**				
Congestive heart failure	769 (38.43%)	574 (36.44%)	195 (45.77%)	<0.001
Cerebrovascular disease	142 (7.10%)	113 (7.17%)	29 (6.81%)	0.793
Peripheral vascular disease	200 (10.00%)	153 (9.71%)	47 (11.03%)	0.421
Dementia	70 (3.50%)	53 (3.37%)	17 (3.99%)	0.533
Chronic pulmonary disease	441 (22.04%)	313 (19.87%)	128 (30.05%)	<0.001
Rheumatic disease	89 (4.45%)	56 (3.56%)	33 (7.75%)	<0.001
Peptic ulcer disease	20 (1.00%)	15 (0.95%)	5 (1.17%)	0.684
Mild liver disease	79 (3.95%)	53 (3.37%)	26 (6.10%)	0.010
Diabetes without complication	524 (26.19%)	403 (25.59%)	121 (28.40%)	0.241
Diabetes with complication	273 (13.64%)	196 (12.44%)	77 (18.08%)	0.003
Paraplegia	14 (0.70%)	12 (0.76%)	2 (0.47%)	0.521
Renal disease	473 (23.64%)	320 (20.32%)	153 (35.92%)	<0.001
Malignant cancer	104 (5.20%)	84 (5.33%)	20 (4.69%)	0.598
Severe liver disease	9 (0.45%)	4 (0.25%)	5 (1.17%)	0.025
Metastatic solid tumor	36 (1.80%)	30 (1.90%)	6 (1.41%)	0.494
AIDS	11 (0.55%)	7 (0.44%)	4 (0.94%)	0.221

*^†^Maximum value within 24 h after admitted to the emergency department.*

*^‡^Calculated or identified according to diagnoses records during the hospitalization.*

*CK-MB, Creatine kinase, MB isoenzyme; PTCA, percutaneous transluminal coronary angioplasty; CABG, coronary artery bypass graft; AIDS, Acquired immunodeficiency syndrome.*

### Association Between Pre-existing Proton Pump Inhibitor Use and the Study Outcomes

As presented in Table 2, there was no statistically significant difference in hospital mortality (4.23% vs. 5.46%, *P* = 0.308) and length of (total) ICU stay [2.07 (1.32–3.82) vs. 1.79 (1.08–3.43) days, *P* = 0.126] between the two cohorts, but patients with pre-existing PPI use showed a statistically significantly longer length of hospital stay [3.81 (2.48–6.91) vs. 3.20 (2.14–5.79) days, *P* = 0.002] and lower proportion of being admitted to ICU (25.59% vs. 40.83%, *P* < 0.001) compared to those without pre-existing PPI use. Regarding types of (first) ICU admission was observed between the two cohorts, patients without pre-existing PPI use had higher proportion of being admitted to coronary care unit (61.28% vs. 55.05%), although the difference was not statistically significant.

**TABLE 2:** Prognosis of the study population according to pre-existing use of proton pump inhibitor.

	Pre-existing use of proton pump inhibitor		*P*
	No (*n* = 1,575)	Yes (*n* = 426)	
Hospital mortality	86 (5.46%)	18 (4.23%)	0.308
Length of hospital stay (days)	3.20 (2.14–5.79)	3.81 (2.48–6.91)	0.002
Being admitted to ICU	643 (40.83%)	109 (25.59%)	<0.001
Type of (first) ICU admission			0.052
Coronary care unit (CCU)	394 (61.28%)	60 (55.05%)	
Cardiac vascular intensive care unit (CVICU)	178 (27.68%)	30 (27.52%)	
Medical intensive care unit (MICU)/Surgical intensive care unit (SICU)/Medical/surgical intensive care unit (MICU/SICU)	57 (8.86%)	17 (15.60%)	
Trauma SICU (TSICU)	11 (1.71%)	0 (0.00%)	
Neuro surgical intensive care unit (Neuro SICU)/Neuro Stepdown	3 (0.47%)	2 (1.83%)	
Length of (total) ICU stay (days)	1.79 (1.08–3.43)	2.07 (1.32–3.82)	0.126

*ICU, intensive care unit.*

The associations of the covariates with the studied outcomes were presented in Supplementary Tables 2–5. After adjusted for potential confounding, results of multivariable analyses (Table 3) indicated pre-existing PPI use was not associated with hospital mortality [odds ratio (OR) 1.08, 95% confidence interval (CI) 0.58–1.99, *P* = 0.818], length of hospital stay (β = 0.23, 95% CI −0.35 to 0.82, *P* = 0.436), and length of (total) ICU stay (β = −0.18, 95% CI −1.06 to 0.69, *P* = 0.680), but was statistically significantly associated with lower risk of being admitted to ICU (OR 0.69, 95% CI 0.52–0.92, *P* = 0.011).

**TABLE 3:** Associations of pre-existing use of proton pump inhibitor and prognosis of the study population.

	Crude			Adjusted^†^		
	Odds ratio (or β)	95% CI	*P*	Odds ratio (or β)	95% CI	*P*
**Hospital mortality**						
**Pre-existing use of proton pump inhibitor**						
No	1 (Reference)			1 (Reference)		
Yes	0.76	0.45–1.28	0.310	1.08	0.58–1.99	0.818
**Length of hospital stay (days)**						
**Pre-existing use of proton pump inhibitor**						
No	0 (Reference)			0 (Reference)		
Yes	0.75	0.10–1.41	0.025	0.23	−0.35 to 0.82	0.436
**Being admitted to ICU**						
**Pre-existing use of proton pump inhibitor**						
No	1 (Reference)			1 (Reference)		
Yes	0.50	0.39–0.63	<0.001	0.69	0.52–0.92	0.011
**Length of (total) ICU stay (days)**						
**Pre-existing use of proton pump inhibitor**						
No	0 (Reference)			0 (Reference)		
Yes	0.22	−0.69 to 1.12	0.639	−0.18	−1.06 to 0.69	0.680

*^†^For the outcome hospital mortality, age, sex, ethnicity, troponin T, CK-MB, PTCA, dilation of coronary artery, Carlson comorbidity index, congestive heart failure, cerebrovascular disease, peripheral vascular disease, dementia, chronic pulmonary disease, mild liver disease, and renal disease were adjusted for; for the outcome length of hospital stay, age, ethnicity, PTCA, dilation of coronary artery, CABG, Charlson comorbidity index, congestive heart failure, cerebrovascular disease, peripheral vascular disease, chronic pulmonary disease, peptic ulcer disease, mild liver disease, diabetes without complication, diabetes with complication, paraplegia, renal disease, malignant cancer, severe liver disease, and metastatic solid tumor were adjusted for; for the outcome being admitted to ICU, sex, ethnicity, prior H_2_ receptor antagonist, troponin T, CK-MB, PTCA, dilation of coronary artery, Charlson comorbidity index, congestive heart failure, cerebrovascular disease, peripheral vascular disease, peptic ulcer disease, mild liver disease, paraplegia, and renal disease were adjusted for; for the outcome length of (total) ICU stay, ethnicity, prior H_2_ receptor antagonist, PTCA, dilation of coronary artery, CABG, Charlson comorbidity index, congestive heart failure, cerebrovascular disease, peripheral vascular disease, peptic ulcer disease, mild liver disease, and renal disease were adjusted for.*

*CI, confidence interval; ICU, intensive care unit; CK-MB, Creatine kinase, MB isoenzyme; PTCA, percutaneous transluminal coronary angioplasty; CABG, coronary artery bypass graft.*

### Association Between Pre-existing Histamine 2 Receptor Antagonists Use and the Study Outcomes

After adjusted for potential confounding, results of multivariable analyses (Table 4) indicated pre-existing H_2_RA use was not associated with hospital mortality (OR 0.48, 95% CI 0.11–2.04, *P* = 0.317), length of hospital stay (β = −0.20, 95% CI −1.31 to 0.91, *P* = 0.728), being admitted to ICU (OR 0.69, 95% CI 0.40–1.20, *P* = 0.188), but was statistically significantly associated with longer length of (total) ICU stay (β = 1.72, 95% CI 0.10–3.34, *P* = 0.038).

**TABLE 4:** Associations of pre-existing use of H2 receptor antagonist and prognosis of the study population.

	Crude			Adjusted^†^		
	Odds ratio (or β)	95% CI	*P*	Odds ratio (or β)	95% CI	*P*
**Hospital mortality**						
**Pre-existing use of H_2_ receptor antagonist**						
No	1 (Reference)			1 (Reference)		
Yes	0.58	0.18–1.86	0.360	0.48	0.11–2.04	0.317
**Length of hospital stay (days)**						
**Pre-existing use of H_2_ receptor antagonist**						
No	0 (Reference)			0 (Reference)		
Yes	0.78	−0.49 to 2.05	0.227	−0.20	−1.31 to 0.91	0.728
**Being admitted to ICU**						
**Pre-existing use of H_2_ receptor antagonist**						
No	1 (Reference)			1 (Reference)		
Yes	0.63	0.40–0.99	0.045	0.69	0.40–1.20	0.188
**Length of (total) ICU stay (days)**						
**Pre-existing use of H_2_ receptor antagonist**						
No	0 (Reference)			0 (Reference)		
Yes	2.66	0.95–4.36	0.002	1.72	0.10–3.34	0.038

*^†^For the outcome hospital mortality, age, sex, ethnicity, troponin T, CK-MB, PTCA, dilation of coronary artery, Carlson comorbidity index, congestive heart failure, cerebrovascular disease, peripheral vascular disease, dementia, chronic pulmonary disease, mild liver disease, and renal disease were adjusted for; for the outcome length of hospital stay, age, ethnicity, pre-existing use of proton pump inhibitor, PTCA, dilation of coronary artery, CABG, Charlson comorbidity index, congestive heart failure, cerebrovascular disease, peripheral vascular disease, chronic pulmonary disease, peptic ulcer disease, mild liver disease, diabetes without complication, diabetes with complication, paraplegia, renal disease, malignant cancer, severe liver disease, and metastatic solid tumor were adjusted for; for the outcome being admitted to ICU, sex, ethnicity, pre-existing use of proton pump inhibitor, troponin T, CK-MB, PTCA, dilation of coronary artery, Charlson comorbidity index, congestive heart failure, cerebrovascular disease, peripheral vascular disease, peptic ulcer disease, mild liver disease, paraplegia, and renal disease were adjusted for; for the outcome length of (total) ICU stay, ethnicity, PTCA, dilation of coronary artery, CABG, Charlson comorbidity index, congestive heart failure, cerebrovascular disease, peripheral vascular disease, peptic ulcer disease, mild liver disease, and renal disease were adjusted for.*

*CI, confidence interval; ICU, intensive care unit; CK-MB, Creatine kinase, MB isoenzyme; PTCA, percutaneous transluminal coronary angioplasty; CABG, coronary artery bypass graft.*

## Discussion

The current study enrolled about 2,000 patients who were admitted to ED due to AMI and investigated the association between pre-existing PPI use before ED visit and short-term prognosis. The main findings of our study are: (1) pre-existing PPI use before ED visit was not an independent risk factor of worse short-term prognosis of AMI patients; (2) AMI patients who were receiving PPI before admission were less likely to be admitted to ICU compared to those did not; (3) in contrast to PPI, lower risk of ICU admission was not observed in AMI patients with pre-existing H2RA use, which, instead, was associated with longer length of ICU stay. These findings do not support the safety concern of PPI that newly diagnosed AMI patients with pre-existing PPI use before ED visit would experience worse short-term prognosis than those without.

Although the findings are negative (i.e., null), it is clinically relevant to investigate the safety of PPI use in AMI patients for the below reasons. First, PPI is widely used in practice and a substantial proportion of patients (i.e., 21.3% in our study) were already on PPI treatment when developing AMI. Second, all the existing studies only investigated the safety of PPI use which was started after AMI had been established. Third, the benefit of using PPI for stress ulcer prophylaxis in critically ill settings may also apply to AMI patients (32, 33), as stress ulcer and related gastrointestinal bleeding are not rare in AMI patients (34) and early use of PPI may benefit AMI patients by decreasing gastrointestinal bleeding (35). Fourth, both the trials and observational studies revealed that the use of PPI significantly reduced the risks of gastrointestinal bleeding when receiving dual antiplatelet therapy which was indicated for AMI (29).

The safety concern about PPI use in patients with AMI mainly came from the observed increased risk of major adverse cardiovascular events in patients with acute coronary syndromes receiving long-term PPI (24). The potential metabolic interaction between clopidogrel and PPI (36) may result in decreased efficacy of antiplatelet agent and lead to increased risk of major adverse cardiovascular events. These mechanisms may play no role the association we investigated (i.e., PPI started before AMI was established), but there are several other mechanisms proposed (24), including hypomagnesemia caused by chronic PPI use which may promote arrhythmia, adverse effects of treatment for clinically significant vitamin B12 deficiency, increased platelet reactivity and thrombosis due to impaired activity of the enzyme dimethylarginine dimethylaminohydrolase, and endothelial lysosomal acidification impaired by PPI. In addition, it has been reported that PPI treatment alone was also associated with increased risk of adverse cardiovascular effect (37).

However, although the above suspected mechanisms may be valid, the observed increased risk of major adverse cardiovascular events could be also simply due to methodological limitations existed in the available studies. This is supported by the study conducted by Jena et al. (22) in which PPI use was found to be associated with community-acquired pneumonia but also implausibly associated with several common medical conditions, suggesting the observed associations were at high risk of bias (due to confounding). In the recent meta-analysis conducted by Guo et al. (29), patients with coronary artery disease receiving dual antiplatelet therapy were included from 6 randomized controlled trials and 16 observational studies. Analysis of the trials did not find an association between incidences of major adverse cardiovascular events and mortality and PPI use, while inconsistent associations were observed between different types of PPI, supporting confounding may play a major role in the observed increased risk of major adverse cardiovascular events in PPI users.

Compared to these studies, our study has some difference in the study design. First, the exposure we studied was pre-existing PPI use before ED visit, instead of PPI started after AMI had been established. Second, we focused on short-term prognosis (i.e., survival during the hospitalization). Such a design enables our study to provide new evidence about the safety concern about PPI use in AMI patients, because if PPI use does causally lead to poor prognosis of AMI, worse prognosis should also be observed in AMI patients with pre-existing PPI use. In the study we also investigated the association between pre-existing H_2_RA use and the study outcomes, and the observed potential benefit of pre-existing PPI use (i.e., lower risk of ICU admission) was not observed in patients with pre-existing H_2_RA use. This extra investigation severs as an active comparator and suggests the robustness of our finding about pre-existing PPI use.

Some limitations should be noted in our study. First, the study was a retrospective study design, and therefore misclassification cannot be ruled out. For example, information about the indications, durations and dosing of the pre-existing PPI use were unknown, and very a few patients were receiving more than one type of PPI. Second, due to the lack of long-term survival data, we only investigated short-term prognosis as the study outcomes, and the association of pre-existing PPI use with long-term prognosis in AMI patients remains unknown in our study. Third, our findings are at risk of residual confounding given the nature of an observational study design. For example, we did not include use of PPI during the hospitalization into account. However, it seems reasonable to assume patients with pre-existing indications for PPI use were more likely to receive PPI during the hospitalization. In addition, the many covariates we studied and the result from the investigation on pre-existing H_2_RA use suggest the robustness of our findings. Fourth, the sample size we included are not large enough to investigate the association between pre-existing PPI use and the study outcomes according to the specific type of PPI. Last, our findings do not support that AMI patients with pre-existing PPI experienced worse short-term prognosis, and seem to suggest AMI patients may benefit from PPI use as lower risk of ICU admission was observed in our study. However, such an interpretation should be cautious due to the limitation of study design (i.e., a non-randomized design). In our study the patients without pre-existing PPI use had significantly higher proportions of receiving PTCA and dilation of coronary artery than those with pre-existing PPI use, which may, to some extent, explain why they were more likely to be admitted to ICU. These limitations should be considered as the directions of improvement when conducting similar research in the future.

## Conclusion

In conclusion, the current study does not support newly diagnosed AMI patients with pre-existing PPI use before ED visit would experience worse short-term prognosis than those without. Studies with better designs especially randomized controlled trials are warranted to confirm our findings.

## Data Availability Statement

Publicly available datasets were analyzed in this study. This data can be found here: The datasets Medical Information Mart for Intensive Care (MIMIC)-IV (version 1.0) and its module the MIMIC-IV-ED database (version 1.0) for this study can be found in the PhysioNet (https://physionet.org/content/mimiciv/1.0/ and https://physionet.org/content/mimic-iv-ed/1.0/).

## Ethics Statement

The studies involving human participants were reviewed and approved by the Ethics Committee of the First People’s Hospital of Chenzhou. Written informed consent for participation was not required for this study in accordance with the national legislation and the institutional requirements.

## Author Contributions

JX verified the analysis, interpreted the results, and wrote the first draft of the manuscript. QC extracted the data, performed the analyses, and revised the manuscript. DH designed the study, interpreted the results, and revised the manuscript. All authors approved the final version of the manuscript for submission.

## Conflict of Interest

The authors declare that the research was conducted in the absence of any commercial or financial relationships that could be construed as a potential conflict of interest.

## Publisher’s Note

All claims expressed in this article are solely those of the authors and do not necessarily represent those of their affiliated organizations, or those of the publisher, the editors and the reviewers. Any product that may be evaluated in this article, or claim that may be made by its manufacturer, is not guaranteed or endorsed by the publisher.

## Footnotes

1
https://github.com/MIT-LCP/mimic-iv
