# Corrigendum: Pre-existing proton pump inhibitor treatment and short-term prognosis of acute myocardial infarction patients

**DOI:** 10.3389/fcvm.2022.987880

**Published:** 2022-07-20

**Authors:** Juntao Xie, Qingui Chen, Dejian He

**Affiliations:** ^1^Intensive Care Unit, The First People's Hospital of Chenzhou, Chenzhou, China; ^2^Intensive Care Unit, The Chenzhou Affiliated Hospital, Hengyang Medical School, University of South China, Chenzhou, China; ^3^Department of Medical Intensive Care Unit, The First Affiliated Hospital, Sun Yat-sen University, Guangzhou, China; ^4^Department of Emergency, The First People's Hospital of Chenzhou, Chenzhou, China; ^5^Department of Emergency, The Chenzhou Affiliated Hospital, Hengyang Medical School, University of South China, Chenzhou, China

**Keywords:** myocardial infarction, proton pump inhibitors, histamine 2 receptor antagonists, risk factors, prognosis

In the published article, there was an error regarding the affiliation for Juntao Xie and Dejian He. As well as having the affiliation “The Chenzhou Affiliated Hospital, Hengyang Medical School, University of South China,” they should also have “The First People's Hospital of Chenzhou.”

The authors apologize for this error and state that this does not change the scientific conclusions of the article in any way. The original article has been updated.

## Publisher's note

All claims expressed in this article are solely those of the authors and do not necessarily represent those of their affiliated organizations, or those of the publisher, the editors and the reviewers. Any product that may be evaluated in this article, or claim that may be made by its manufacturer, is not guaranteed or endorsed by the publisher.

