# Supplementary material for: Pre-existing Proton Pump Inhibitor Treatment and Short-Term Prognosis of Acute Myocardial Infarction Patients
Source: Front Cardiovasc Med. 2022 Jul 4;9:919716. doi: 10.3389/fcvm.2022.919716 (PMC9289161; doi:10.3389/fcvm.2022.919716)
Supplement: Supplementary file 1 [file Data_Sheet_1.docx]

Supplementary Material

# Supplementary Tables

## Supplementary Table 1 Detailed types of myocardial infarction in the study population

| Codes | Type of ICD codes | Type of myocardial infarction | N (%) |
| --- | --- | --- | --- |
| 410.91 | ICD-9-CM | Acute myocardial infarction of unspecified site, initial episode of care | 644 (72.69%) |
| 410.71 | ICD-9-CM | Subendocardial infarction, initial episode of care | 226 (25.51%) |
| 410.41 | ICD-9-CM | Acute myocardial infarction of other inferior wall, initial episode of care | 6 (0.68%) |
| 410.11 | ICD-9-CM | Acute myocardial infarction of other anterior wall, initial episode of care | 6 (0.68%) |
| 410.31 | ICD-9-CM | Acute myocardial infarction of inferoposterior wall, initial episode of care | 2 (0.23%) |
| 410.50 | ICD-9-CM | Acute myocardial infarction of other lateral wall, episode of care unspecified | 1 (0.11%) |
| 410.92 | ICD-9-CM | Acute myocardial infarction of unspecified site, subsequent episode of care | 1 (0.11%) |
| I21.4 | ICD-10-CM | Non-ST elevation (NSTEMI) myocardial infarction | 817 (73.27%) |
| I21.3 | ICD-10-CM | ST elevation (STEMI) myocardial infarction of unspecified site | 216 (19.37%) |
| I21.19 | ICD-10-CM | ST elevation (STEMI) myocardial infarction involving other coronary artery of inferior wall | 72 (6.46%) |
| I21.09 | ICD-10-CM | ST elevation (STEMI) myocardial infarction involving other coronary artery of anterior wall | 5 (0.45%) |
| I21.29 | ICD-10-CM | ST elevation (STEMI) myocardial infarction involving other sites | 3 (0.27%) |
| I21.9 | ICD-10-CM | Acute myocardial infarction, unspecified | 2 (0.18%) |

Abbreviations: ICD, International Classification of Diseases; ICD-9-CM, ICD-9-Clinical Modification; ICD-10-CM, ICD-10-Clinical Modification.

## Supplementary Table 2 Association of the studied clinical characteristics with hospital mortality according to univariable logistic regression

|  | Odds ratio | 95% CI | P^#^ |
| --- | --- | --- | --- |
| Age (years) | 1.05 | 1.04-1.07 | **<0.0001** |
| Sex |  |  |  |
| Male | 1 (Reference) |  |  |
| Female | 1.45 | 0.97-2.15 | **0.0673** |
| Ethnicity |  |  |  |
| White | 1 (Reference) |  |  |
| Black/African American | 1.31 | 0.69-2.49 | 0.4012 |
| Hispanic/Latino | 0.00 | 0.00-Inf | 0.9769 |
| Asian | 0.81 | 0.19-3.40 | 0.7752 |
| Other/unknown | 3.93 | 2.47-6.24 | **<0.0001** |
| Prior H_2_ receptor antagonist |  |  |  |
| No | 1 (Reference) |  |  |
| Yes | 0.58 | 0.18-1.86 | 0.3597 |
| Troponin T^†^ (ng/mL) | 1.15 | 1.09-1.20 | **<0.0001** |
| CK-MB^†^ (ng/mL) | 1.00 | 1.00-1.01 | **<0.0001** |
| PTCA |  |  |  |
| No | 1 (Reference) |  |  |
| Yes | 0.44 | 0.23-0.83 | **0.0116** |
| Dilation of coronary artery |  |  |  |
| No | 1 (Reference) |  |  |
| Yes | 0.55 | 0.31-0.96 | **0.0340** |
| CABG |  |  |  |
| No | 1 (Reference) |  |  |
| Yes | 0.78 | 0.36-1.71 | 0.5347 |
| Charlson Comorbidity Index^‡^ | 1.20 | 1.12-1.29 | **<0.0001** |
| Comorbidities^‡*^ |  |  |  |
| Congestive heart failure | 2.06 | 1.38-3.07 | **0.0004** |
| Cerebrovascular disease | 3.22 | 1.89-5.47 | **<0.0001** |
| Peripheral vascular disease | 1.70 | 0.98-2.97 | **0.0595** |
| Dementia | 4.66 | 2.46-8.83 | **<0.0001** |
| Chronic pulmonary disease | 1.51 | 0.97-2.35 | **0.0670** |
| Rheumatic disease | 1.40 | 0.59-3.30 | 0.4422 |
| Peptic ulcer disease | 0.97 | 0.13-7.31 | 0.9754 |
| Mild liver disease | 2.22 | 1.04-4.76 | **0.0403** |
| Diabetes without complication | 1.40 | 0.92-2.14 | 0.1157 |
| Diabetes with complication | 1.00 | 0.56-1.79 | 0.9895 |
| Paraplegia | 1.38 | 0.18-10.68 | 0.7560 |
| Renal disease | 1.61 | 1.05-2.48 | **0.0298** |
| Malignant cancer | 1.56 | 0.73-3.29 | 0.2485 |
| Severe liver disease | 2.52 | 0.31-20.54 | 0.3874 |
| Metastatic solid tumor | 1.06 | 0.25-4.48 | 0.9349 |
| AIDS | 0.00 | 0.00-Inf | 0.9770 |

Notes: † Maximum value within 24 hours after admitted to the emergency department

‡ Calculated or identified according to diagnoses records during the hospitalization

* Compared to patients without the comorbidity

# P values <0.1 were shown in bold, which were included in the multivariable analyses

Abbreviations: CI, confidence interval; CK-MB, Creatine kinase, MB isoenzyme; PTCA, percutaneous transluminal coronary angioplasty; CABG, coronary artery bypass graft; AIDS, Acquired immunodeficiency syndrome.

## Supplementary Table 3 Association of the studied clinical characteristics with length of hospital stay (days) according to univariable linear regression

|  | β | 95% CI | P^#^ |
| --- | --- | --- | --- |
| Age (years) | 0.03 | 0.01 to 0.05 | **0.0011** |
| Sex |  |  |  |
| Male | 0 (Reference) |  |  |
| Female | -0.07 | -0.61 to 0.48 | 0.8113 |
| Ethnicity |  |  |  |
| White | 0 (Reference) |  |  |
| Black/African American | -0.20 | -1.06 to 0.66 | 0.6504 |
| Hispanic/Latino | -1.37 | -2.91 to 0.18 | **0.0831** |
| Asian | 0.08 | -1.50 to 1.66 | 0.9197 |
| Other/unknown | -0.80 | -1.68 to 0.07 | **0.0703** |
| Prior H_2_ receptor antagonist |  |  |  |
| No | 0 (Reference) |  |  |
| Yes | 0.78 | -0.49 to 2.05 | 0.2271 |
| Troponin T^†^ (ng/mL) | 0.05 | -0.04 to 0.15 | 0.2679 |
| CK-MB^†^ (ng/mL) | -0.00 | -0.00 to 0.00 | 0.7719 |
| PTCA |  |  |  |
| No | 0 (Reference) |  |  |
| Yes | -1.49 | -2.16 to -0.82 | **<0.0001** |
| Dilation of coronary artery |  |  |  |
| No | 0 (Reference) |  |  |
| Yes | -1.28 | -1.92 to -0.65 | **<0.0001** |
| CABG |  |  |  |
| No | 0 (Reference) |  |  |
| Yes | 8.34 | 7.44 to 9.24 | **<0.0001** |
| Charlson Comorbidity Index^‡^ | 0.64 | 0.54 to 0.73 | **<0.0001** |
| Comorbidities^‡*^ |  |  |  |
| Congestive heart failure | 3.24 | 2.71 to 3.77 | **<0.0001** |
| Cerebrovascular disease | 3.58 | 2.55 to 4.61 | **<0.0001** |
| Peripheral vascular disease | 1.39 | 0.50 to 2.28 | **0.0023** |
| Dementia | -0.15 | -1.61 to 1.31 | 0.8389 |
| Chronic pulmonary disease | 1.35 | 0.70 to 2.00 | **<0.0001** |
| Rheumatic disease | -0.74 | -2.05 to 0.56 | 0.2635 |
| Peptic ulcer disease | 9.45 | 6.79 to 12.12 | **<0.0001** |
| Mild liver disease | 3.17 | 1.80 to 4.54 | **<0.0001** |
| Diabetes without complication | 1.10 | 0.49 to 1.71 | **0.0004** |
| Diabetes with complication | 2.73 | 1.96 to 3.51 | **<0.0001** |
| Paraplegia | 6.05 | 2.84 to 9.26 | **0.0002** |
| Renal disease | 2.06 | 1.43 to 2.69 | **<0.0001** |
| Malignant cancer | 1.97 | 0.76 to 3.18 | **0.0014** |
| Severe liver disease | 4.83 | 0.82 to 8.84 | **0.0184** |
| Metastatic solid tumor | 2.31 | 0.30 to 4.33 | **0.0246** |
| AIDS | 2.64 | -0.99 to 6.27 | 0.1540 |

Notes: † Maximum value within 24 hours after admitted to the emergency department

‡ Calculated or identified according to diagnoses records during the hospitalization

* Compared to patients without the comorbidity

# P values <0.1 were shown in bold, which were included in the multivariable analyses

Abbreviations: CI, confidence interval; CK-MB, Creatine kinase, MB isoenzyme; PTCA, percutaneous transluminal coronary angioplasty; CABG, coronary artery bypass graft; AIDS, Acquired immunodeficiency syndrome.

## Supplementary Table 4 Association of the studied clinical characteristics with being admitted to ICU according to univariable logistic regression

|  | Odds ratio | 95% CI | P^#^ |
| --- | --- | --- | --- |
| Age (years) | 1.00 | 0.99-1.01 | 0.6766 |
| Sex |  |  |  |
| Male | 1 (Reference) |  |  |
| Female | 0.81 | 0.67-0.98 | **0.0266** |
| Ethnicity |  |  |  |
| White | 1 (Reference) |  |  |
| Black/African American | 1.00 | 0.75-1.34 | 0.9906 |
| Hispanic/Latino | 0.68 | 0.38-1.19 | 0.1773 |
| Asian | 1.29 | 0.76-2.19 | 0.3475 |
| Other/unknown | 2.04 | 1.53-2.72 | **<0.0001** |
| Prior H_2_ receptor antagonist |  |  |  |
| No | 1 (Reference) |  |  |
| Yes | 0.63 | 0.40-0.99 | **0.0450** |
| Troponin T^†^ (ng/mL) | 1.46 | 1.37-1.55 | **<0.0001** |
| CK-MB^†^ (ng/mL) | 1.01 | 1.01-1.01 | **<0.0001** |
| PTCA |  |  |  |
| No | 1 (Reference) |  |  |
| Yes | 1.77 | 1.42-2.21 | **<0.0001** |
| Dilation of coronary artery |  |  |  |
| No | 1 (Reference) |  |  |
| Yes | 0.74 | 0.59-0.92 | **0.0067** |
| CABG |  |  |  |
| No | 1 (Reference) |  |  |
| Yes | inf. | 0.00-inf. | 0.9515 |
| Charlson Comorbidity Index^‡^ | 1.09 | 1.05-1.13 | **<0.0001** |
| Comorbidities^‡*^ |  |  |  |
| Congestive heart failure | 1.99 | 1.65-2.40 | **<0.0001** |
| Cerebrovascular disease | 2.30 | 1.62-3.26 | **<0.0001** |
| Peripheral vascular disease | 1.34 | 1.00-1.81 | **0.0523** |
| Dementia | 0.94 | 0.57-1.55 | 0.8019 |
| Chronic pulmonary disease | 0.99 | 0.79-1.24 | 0.9516 |
| Rheumatic disease | 0.98 | 0.63-1.55 | 0.9438 |
| Peptic ulcer disease | 2.63 | 1.06-6.54 | **0.0371** |
| Mild liver disease | 1.72 | 1.09-2.73 | **0.0198** |
| Diabetes without complication | 1.18 | 0.96-1.45 | 0.1240 |
| Diabetes with complication | 1.17 | 0.89-1.52 | 0.2578 |
| Paraplegia | 9.96 | 2.21-44.98 | **0.0028** |
| Renal disease | 1.24 | 1.00-1.54 | **0.0550** |
| Malignant cancer | 0.78 | 0.51-1.20 | 0.2586 |
| Severe liver disease | 0.59 | 0.12-2.89 | 0.5132 |
| Metastatic solid tumor | 1.30 | 0.67-2.54 | 0.4410 |
| AIDS | 1.05 | 0.30-3.65 | 0.9394 |

Notes: † Maximum value within 24 hours after admitted to the emergency department

‡ Calculated or identified according to diagnoses records during the hospitalization

* Compared to patients without the comorbidity

# P values <0.1 were shown in bold, which were included in the multivariable analyses

Abbreviations: ICU, intensive care unit; CI, confidence interval; CK-MB, Creatine kinase, MB isoenzyme; PTCA, percutaneous transluminal coronary angioplasty; CABG, coronary artery bypass graft; AIDS, Acquired immunodeficiency syndrome.

## Supplementary Table 5 Association of the studied clinical characteristics with length of (total) ICU stay (days) among patients with ICU admissions according to univariable linear regression

|  | β | 95% CI | P^#^ |
| --- | --- | --- | --- |
| Age (years) | 0.01 | -0.01 to 0.04 | 0.2196 |
| Sex |  |  |  |
| Male | 0 (Reference) |  |  |
| Female | -0.28 | -0.94 to 0.38 | 0.3994 |
| Ethnicity |  |  |  |
| White | 0 (Reference) |  |  |
| Black/African American | -0.88 | -1.93 to 0.16 | **0.0985** |
| Hispanic/Latino | -1.05 | -3.20 to 1.11 | 0.3409 |
| Asian | 0.67 | -1.12 to 2.45 | 0.4663 |
| Other/unknown | -0.65 | -1.53 to 0.24 | 0.1527 |
| Prior H_2_ receptor antagonist |  |  |  |
| No | 0 (Reference) |  |  |
| Yes | 2.66 | 0.95 to 4.36 | **0.0023** |
| Troponin T^†^ (ng/mL) | 0.01 | -0.07 to 0.09 | 0.8360 |
| CK-MB^†^ (ng/mL) | -0.00 | -0.00 to 0.00 | 0.7693 |
| PTCA |  |  |  |
| No | 0 (Reference) |  |  |
| Yes | -0.86 | -1.59 to -0.14 | **0.0197** |
| Dilation of coronary artery |  |  |  |
| No | 0 (Reference) |  |  |
| Yes | -0.83 | -1.63 to -0.03 | **0.0414** |
| CABG |  |  |  |
| No | 0 (Reference) |  |  |
| Yes | 2.55 | 1.80 to 3.30 | **<0.0001** |
| Charlson Comorbidity Index^‡^ | 0.31 | 0.19 to 0.43 | **<0.0001** |
| Comorbidities^‡*^ |  |  |  |
| Congestive heart failure | 1.50 | 0.87 to 2.13 | **<0.0001** |
| Cerebrovascular disease | 2.06 | 1.04 to 3.08 | **<0.0001** |
| Peripheral vascular disease | 0.88 | -0.11 to 1.88 | **0.0825** |
| Dementia | -0.88 | -2.66 to 0.90 | 0.3305 |
| Chronic pulmonary disease | 0.20 | -0.58 to 0.98 | 0.6200 |
| Rheumatic disease | -0.25 | -1.85 to 1.36 | 0.7644 |
| Peptic ulcer disease | 5.33 | 2.82 to 7.84 | **<0.0001** |
| Mild liver disease | 1.81 | 0.36 to 3.26 | **0.0146** |
| Diabetes without complication | 0.45 | -0.26 to 1.16 | 0.2170 |
| Diabetes with complication | 0.59 | -0.33 to 1.50 | 0.2099 |
| Paraplegia | 1.40 | -1.14 to 3.94 | 0.2813 |
| Renal disease | 1.11 | 0.37 to 1.85 | **0.0035** |
| Malignant cancer | -0.62 | -2.15 to 0.91 | 0.4280 |
| Severe liver disease | -1.06 | -7.24 to 5.13 | 0.7382 |
| Metastatic solid tumor | 1.50 | -0.71 to 3.70 | 0.1836 |
| AIDS | -1.36 | -5.76 to 3.03 | 0.5427 |

Notes: † Maximum value within 24 hours after admitted to the emergency department

‡ Calculated or identified according to diagnoses records during the hospitalization

* Compared to patients without the comorbidity

# P values <0.1 were shown in bold, which were included in the multivariable analyses

Abbreviations: ICU, intensive care unit; CI, confidence interval; CK-MB, Creatine kinase, MB isoenzyme; PTCA, percutaneous transluminal coronary angioplasty; CABG, coronary artery bypass graft; AIDS, Acquired immunodeficiency syndrome.
